# Starting with the End in Mind: Recommendations to Optimize Implementation of a Novel TBI Classification from the 2024 NINDS TBI Classification and Nomenclature Workshop’s Knowledge to Practice Working Group

**DOI:** 10.1089/neu.2024.0576

**Published:** 2025-07-09

**Authors:** Peter Bragge, Molly McNett, Mark Bayley, Maureen Dobbins, Risa Nakase-Richardson, Corinne Peek-Asa, Alexis F. Turgeon, Hibah Awwad, Kristen Dams-O’Connor, Adele Doperalski, Andrew Maas, Mike McCrea, Nsini Umoh, Geoff Manley

**Affiliations:** ^1^ Monash Sustainable Development Institute and Behaviour Works Australia, Monash University, Melbourne, Australia.; ^2^ College of Nursing, Helene Fuld Health Trust National Institute for Evidence-based Practice in Nursing & Healthcare, The Ohio State University, Columbus, Ohio, USA.; ^3^ UHN-Toronto Rehabilitation Institute and Division of Physical Medicine and Rehabilitation, Temerty Faculty of Medicine, University of Toronto, Toronto, Canada.; ^4^ McMaster University, Hamilton, Canada.; ^5^ James A. Haley Veterans Hospital & Department of Internal Medicine, Division of Pulmonary, Critical Care, and Sleep Medicine, University of South Florida, Tampa, Florida, USA.; ^6^ University of California, San Diego, La Jolla, California, USA.; ^7^ Department of Anesthesiology and Critical Care Medicine, Division of Critical Care Medicine, Faculty of Medicine, Université Laval, Québec City, Canada.; ^8^ Population Health and Optimal Health Practice Research Unit, Centre Hospitalier Universitaire de Québec, Université Laval Research Center, Québec City, Canada.; ^9^ Division of Neuroscience, National Institute of Neurological Disorders and Stroke, Bethesda, Maryland, USA.; ^10^ Department of Rehabilitation and Human Performance, Icahn School of Medicine, Mount Sinai, New York, New York, USA.; ^11^ Department of Neurology, Icahn School of Medicine, Mount Sinai, New York, New York, USA.; ^12^ Division of Neuroscience, National Institute of Neurological Disorders and Stroke, Bethesda, Maryland, USA.; ^13^ Department of Neurosurgery, Antwerp University Hospital, Edegem, Belgium.; ^14^ Department of Translational Neuroscience, Faculty of Medicine and Health Science, University of Antwerp, Antwerp, Belgium.; ^15^ Department of Neurosurgery, Medical College of Wisconsin, Milwaukee, Wisconsin, USA.; ^16^ Neurological Surgery, University of California San Francisco, San Francisco, California, USA.

**Keywords:** behavior change, CBI-M, characterization, classification, framework, integrated knowledge translation, NIH, NINDS, nomenclature, prioritization, traumatic brain injury

## Abstract

The Knowledge to Practice Working Group (K2P WG) was one of six expert groups convened in early 2023 to plan the 2024 National Institute of Neurological Disorders and Stroke Traumatic brain injury (TBI) Classification and Nomenclature Workshop. Recognizing that implementation of revised classification systems is essential to achieve intended impact, the K2P WG’s key aims were to foster shared understanding of knowledge translation (KT), build capacity for implementation of a revised TBI classification system, identify and prioritize KT actions, implementation steps and audiences; and make recommendations to advance implementation. The cornerstone of this work was a focused survey to identify “who needs to do what differently,” while prioritizing potential implementation actions. Survey findings, dialogue with other working groups, stakeholder discussions, and public feedback were also utilized to support implementation of the revised Clinical, Biomarker, Imaging-Modifiers and retrospective TBI classification system. Forty researchers across five working groups responded to the survey (Response Rate = 59.7%). Fifty-two unique implementation actions were identified. The top 15 priorities across the five working groups comprised six pertaining to clinical practice (e.g., change Glasgow Coma Scale [GCS] assessment); seven focusing on research (e.g., develop tools for measuring psychological and environmental factors); and one each on lived experience (simplified language for patients and families) and other settings (insurance company support for biomarker testing). Twenty-seven stakeholder groups and 18 target settings were identified as being most impacted by the revised classification system. Key recommendations included: develop guidelines based on systematic reviews, clearly explain the rationale for the change, develop implementation toolkits with input from all stakeholders, and embed the new classification in a learning health system database to facilitate implementation strategies based on audits, feedback, and cost-effectiveness analyses.

## Introduction

Traumatic brain injury (TBI) remains a major global challenge, with an incidence of 50 million per year and a large prevalence of individuals living with TBI symptoms and sequelae.^1^ TBI has a broad array of consequences ranging from temporary to life-altering and this is reflected by considerable associated global economic costs.^1^ Due to the heterogeneity of TBI presentation, accurate classification of TBI is pivotal to optimizing the selection of appropriate health care interventions to maximize rehabilitation and recovery. The current strategy of using the Glasgow Coma Scale (GCS) to define mild, moderate and severe TBI leaves much to be desired in terms of prognostic value.

TBI classification was the focus of a workshop convened in October 2007, which identified research and clinical priorities pertaining to pathophysiologic mechanisms and injury processes, diagnostic tools, prognostic modelling, and outcome assessment.^2^ Building on progress made in these areas, the National Institute of Neurological Disorders and Stroke (NINDS) convened a workshop in January 2024 with a focus on consolidating current knowledge on TBI classification to build a more precise and evidence-based classification system.^3^

In parallel with advances in TBI research since 2007, the science of knowledge translation (KT) has gained prominence. Despite considerable advances in medical research, studies in the early 2000s established that 30–45% of patients do not receive best evidence-based care and 20–25% receive care that is unnecessary or harmful.^4^ KT emerged from this recognition that passive dissemination of research findings is insufficient to drive meaningful change in practice and policy. KT draws upon behavioral, organizational, and psychological sciences to explore and understand individual, institutional, and system-level barriers to practice change and tailor behavior change strategies accordingly.^5–8^ Studies have demonstrated that this approach can aid in reducing the considerable gaps between evidence and practice.^9^

The organizers of the updated TBI classification workshop recognized both the challenge of the change needed for implementation of any new classification system and the emergence of KT science as an avenue for addressing this challenge. Therefore, the implementation science group, subsequently named the *knowledge to practice working group (K2P WG),* was tasked with working alongside the five other WGs developing the updated TBI classification system to keep “the end users in mind” and ensure that implementation was planned from the outset. These five groups comprised global experts on clinical symptoms; imaging; blood-based biomarkers; psychosocial and environmental modifiers; and retrospective classification. These WGs reviewed the science pertaining to these areas and developed elements of the TBI classification system accordingly. Findings of these groups are published across five manuscripts.

This article focuses on the application of KT science to implementation of the proposed new TBI classification framework into policy and practice. The overarching objectives of the K2P WG were:

1. Foster a shared understanding between all elements of the program of key principles of KT science with a particular focus on how KT could meaningfully impact design of the classification system, data collection, and other processes; and
2. Identify and prioritize program elements that could become the focus of active implementation efforts (in addition to routine passive dissemination of reports, presentations, and academic publications).

## Methods

The K2P WG was formed at the instigation of the 2024 TBI Classification and Nomenclature Workshop Steering Committee based on previous collaboration with the lead author (P.B.),^1^ and known contacts in KT science. The Committee worked with PB to expand on this initial list and make enquiries to potential WG members regarding interest and availability. The WG comprised seven members across three countries (Australia, Canada, USA) with experience in applied KT science in TBI/traumatic injury and clinical qualifications in medicine, nursing, neuropsychology, and physical therapy.

### Working group objective 1: Fostering a shared understanding of KT science

**FIG. 1.**
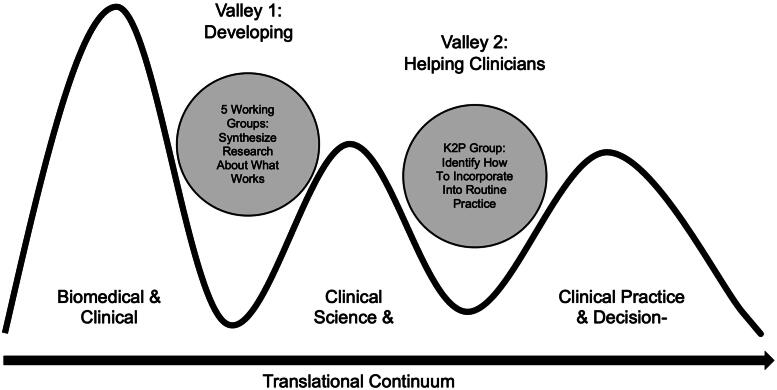
“Valleys of death” in knowledge translation (adapted from Reis, 2008).^10^

The K2P WG was conceived as a “cross cutting” methodological group that interacted with the other five working groups to bring together an implementation agenda for the workshop. Reflecting this, the team developed and delivered a presentation to all working group chairs in July 2023. The first key conceptual underpinning of the K2P working group is displayed in Figure 1, which drew upon previously published approaches to simplifying the representation of KT science.^10,11^ This conveyed the intention to work collaboratively with the other working groups to bridge “valleys of death” in the KT continuum. Each “valley of death” refers to a gap between research evidence and current practice that inhibits or delays translation of research knowledge into clinical practice settings and health care decision-making.^10^ The first step in KT work is to identify and synthesize the evidence that provides an impetus for the practice change or recommendation.^9^ This work, represented by “Valley 1,” reflected the efforts among the other five classification working groups to critically synthesize existing research literature to generate proposed practice recommendations. The work of the K2P WG addresses “Valley 2” and included collaborating with other working group members and key stakeholders in the broader TBI community to identify optimal methods to facilitate use of this information in routine practice settings.

In order to effectively address these “valleys of death,” the second key conceptual underpinning of the initial work of our group was to introduce the concept of integrated knowledge translation, *“a model of collaborative research, where researchers work with knowledge users who identify a problem and have the authority to implement the research recommendations”* [p. 299]^12^ Applying this definition, the *researchers* were members of the K2P WG, and the *knowledge users* included change champions for the new classification system (members of the five working groups), as well as diverse TBI collaborative partners affected by the proposed changes such as clinicians, individuals with lived experience, family caregivers, policy-makers, insurers, and professional societies. The group drew upon the Knowledge to Action Framework^4^ to conceptualize the overall process of information synthesis, dissemination, exchange, and application among these collective groups to improve health care and services.

### Working group objective 2: Identifying and prioritizing determinants to implement a new system of TBI classification into policy and practice

**FIG. 2.**
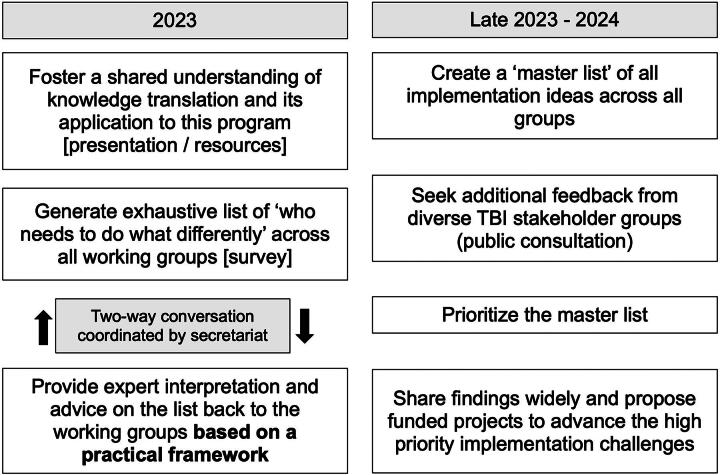
Overview of the K2P WG approach. K2P WG, The Knowledge to Practice Working Group.

Figure 2 summarizes the overall approach of the K2P WG, which drew upon the theories outlined and presented to the other working groups. Drawing upon previously published prioritization methodologies,^13,14^ the approach centered on identification and prioritization of specific target behaviors and practices that would need to change to implement a TBI classification system, as well as identification of critical stakeholders most impacted by these proposed changes.

To generate a comprehensive list of “who needs to do what differently” based on their proposed recommendations, the K2P group created an electronic survey that was administered to each working group between October and December 2023. The survey was developed based on previous prioritization activities and approaches.^13,15^ A draft survey was circulated to the chairs of each working group for comments and refinements were made accordingly. To explain the survey goals and to capture information directly, a K2P working group member attended sessions with each of the other working groups. Notes from these meetings, as well as individual and working group completion of the survey, comprised the response set.

The survey was comprised of six questions that working groups were asked to complete as they developed their proposed recommendations for the workshop. In addition to generating a comprehensive list of “who needs to do what differently,” the survey gathered information on target audiences for each working groups’ recommendations (e.g., emergency department [ED] physicians, community physicians, neurosurgeons, hospital administrators, insurers); settings most impacted by the proposed recommendations, potential change champions, and specific behaviors that would need to occur to successfully integrate proposed changes into practice settings (Appendix A).

Survey results were analyzed in aggregate, and then specifically for each group to create a “master list” of proposed “who needs to do what differently” actions that could influence routine use of proposed recommendations among each working group. These findings were shared with the members of the working groups and each group was then instructed to identify their group’s top three actions.

### Workshop activities

The TBI Classification and Nomenclature Workshop was held in Washington on January 22–23, 2024. A series of roundtable discussions were held in parallel. In addition to hosting a dedicated roundtable, members of the K2P WG attended the other five roundtables, reflecting the cross-cutting purpose of the K2P WG to provide KT input and perspectives to further the ambitions of the other working groups. A brief guide was developed to aid in facilitating this discussion (Appendix B). On day two of the workshop, all working groups reconvened to discuss reflections from the breakout sessions and formulate a list of proposed recommendations informed by these discussions. These were then presented back to all workshop participants in the plenary, followed by plenary discussion and reflection on key workshop activities, findings, and future directions.

Following the workshop, all working groups were asked to discuss and refine their recommendations, incorporating any relevant feedback from the workshop participants and the open public commentary period. These recommendations were the final output of each working group.

## Results

There was a total of 40 responses to the implementation survey from the five working groups, who collectively had 67 members (response rate = 59.7%). Within these responses, there were 108 statements on “who needs to do what differently” in response to the working group’s proposed recommendations, which resulted in 52 unique actions across collaborative partner groups.

### Target groups

**FIG. 3.**
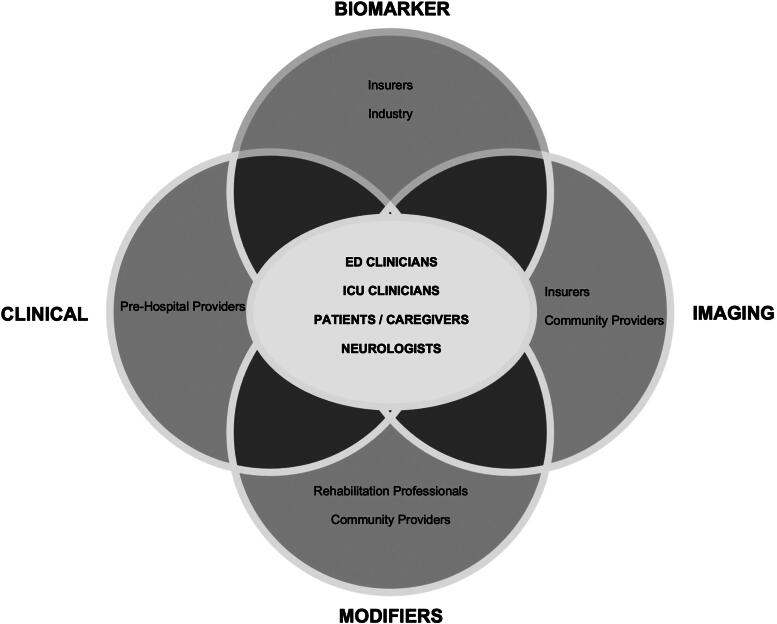
Initial target audiences for proposed CBI-M pillars across working groups. CBI-M, Clinical, Biomarker, Imaging and Modifier.

There were 27 different target audiences or collaborative partner groups initially identified for the proposed changes in the classification scheme. The most frequently identified groups across the proposed Clinical, Biomarker, Imaging and Modifier (CBI-M) pillars included ED physicians, intensive care unit (ICU) physicians, patients/caregivers and neurologists. Additional potential target groups specific for each working group were also identified (Figure 3). The retrospective classification working group shared some common collaborative partners, such as researchers, neurologists, community providers, and insurers, but also identified rehabilitation clinicians and social services personnel.

### Target settings

**FIG. 4.**
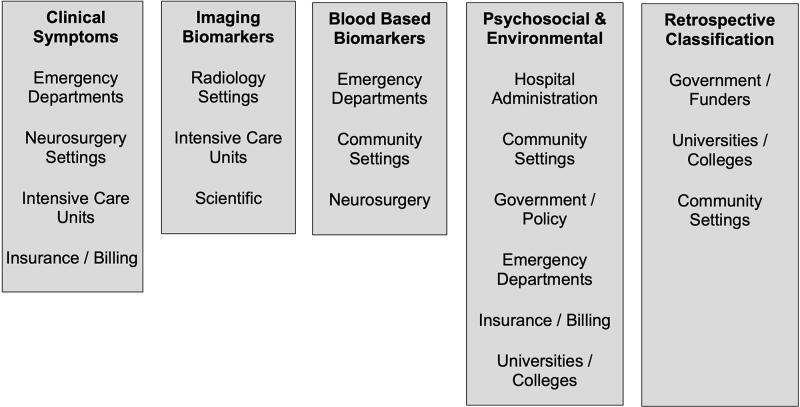
Initial settings most impacted by proposed changes of the working groups.

The working groups also identified various settings most likely to be initially affected by their proposed recommendations. A total of 18 different settings were identified, with frequently identified settings including community health, emergency and intensive care, and university settings (Fig. 4).

### Change champions

Each working group also identified potential change champions, who are individuals or groups that would be instrumental in facilitating uptake of the proposed recommendations into practice.^16^ A total of 14 potential groups of champions were identified, which included: advocacy groups, community or consumer organizations, pre-hospital, hospital, and rehabilitation groups, industry partners, insurance organizations, media outlets, journal publishers, patients/caregivers, policy makers, medical/professional societies, and research societies.

### Specific behavior changes

**FIG. 5.**
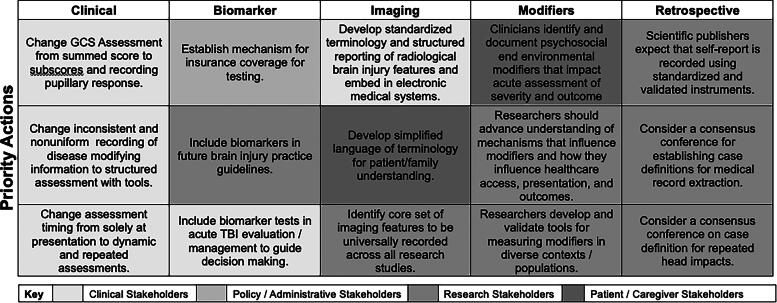
Initial priority actions to support translation of recommendations into practice.

Working group members identified 52 different unique actions that would need to occur to facilitate uptake of proposed recommendations. Each group was then asked to identify the top three priority actions that would need to occur to optimize successful implementation of their recommendations. Figure 5 displays the top three priority areas for each group. The working group action items are shaded to indicate those focused on clinicians, policymakers, patient/caregivers, or researchers.

## Summary of Recommendations

Upon conclusion of the workshop, the K2P WG integrated information from diverse collaborative partner groups that included members of the working groups, the steering committee, workshop attendees, and the broader public (through open commentary feedback) to generate the following recommendations. Short term, initial priority actions to promote translation of the CBI-M pillars and retrospective classification into practice are listed below, mapped to an example from the initial priority actions: *“Change GCS assessment from summed score to subscores and recording pupillary response”*:

• Develop clinical practice guidelines underpinned by systematic reviews as a cornerstone for development of future implementation toolkits. *For example, guidelines for GCS assessment following TBI updated to emphasise recording of subscores and pupillary response, incorporating an evidence grade and scientific rationale.*
• Provide clarity on the problem that is being addressed by the CBI-M pillars and retrospective classification recommendations to generate enthusiasm and buy in for proposed changes (i.e., many collaborative partners will want to truly understand the “why” behind the practice change). *This may involve embarking on an education/information campaign with ED doctors/clinicians and other affected groups (hospital administrators, insurers) demonstrating limitations and risks associated with current GCS practice and the value and importance of recording GCS subscores and pupillary response.*
• Consistent with KT principles and the Knowledge to Action cycle, emphasize integration of the TBI characterization framework with the CBI-M pillars and retrospective classification recommendations as an iterative learning process as part of ongoing work plans. In this collaborative approach, researchers work with knowledge users and key stakeholders to learn from ongoing attempts to implement the classification and revise the approach accordingly. *In the case of updating GCS practices, this could involve working with hospitals to implement changes to electronic medical record (EMR) systems to enable recording of GCS subscores and pupillary responses, including identification of barriers to implementation (e.g., IT issues, end user feedback on useability) and strategies to address them.*
• Engage with collaborative partner groups to develop implementation toolkits that are based on evidence and implementation science principles to support practice changes in alignment with CBI-M pillars and retrospective classification recommendations. Toolkits should include plain language to optimize buy in, adaptability for diverse settings with variable resources, target audiences, short- and long-term incremental actions to support the practice change, and methods for evaluation of implementation efforts. *This could involve packaging GCS guidelines, educational/information materials and “how do” guides to upgrading EMR systems in various settings and systems.*
• Establish learning health systems^17^ that audit, validate, and prospectively evaluate the CBI-M pillars and retrospective classifications using existing datasets to evaluate current practices. *An EMR system could be audited to determine adherence to the new GCS protocol.*
• Incorporate cost effectiveness measures into ongoing and future research evaluating CBI-M pillars and retrospective classification systems to demonstrate the benefit of the system. *A longitudinal study examining prognostic, clinical, and qualitative outcomes pre- and post-implementation of the new GCS protocol could quantify return on investment in this change.*

## Limitations

First, our survey was not validated prior to being administered, including for clinical sensibility, reliability, and face validity. The questionnaire may not have covered all aspects facilitating the implementation of the findings, and questions may have been interpreted differently depending on the respondents. Second, despite efforts to solicit perspectives from a diverse group of collaborative partners and recipients of a revised classification system for TBI, there are limitations to our work and the proposed recommendations. The initially identified partners, settings, and champions identified as part of our work are not exclusive to these groups nor do they represent an exhaustive list of those impacted by the proposed changes to TBI classification. Although the working groups were comprised of leading international experts and feedback was actively solicited from the broader community including individuals with lived experience, given the broad collaborative partner groups involved in optimizing TBI care, representation was not complete. Similarly, this working group included different areas of implementation science expertise but could not represent all TBI potential partners. Identification of these components are only an initial starting point for translational efforts. It is likely that many more individuals and groups will be impacted by the proposed classification system, and these may be identified and engaged through use the iterative knowledge/translation cycle that is proposed. On the other hand, the initial priority actions we report on are those identified by working group members with feedback from workshop participants. As such, they are subject to bias from those individuals responsible for developing the proposed classification system, and do not reflect the full scope of actions that will be required to successfully implement the proposed practice changes associated with a new classification system. Lastly, the proposed practice changes and our recommendations on implementation may not be applicable across all settings or groups. As an example, in countries with universal health coverage, insurance will not be a barrier to implementation, but rather the level of evidence to justify practice changes. We also acknowledge that resources vary considerably across settings and groups, and these factors will likely have substantial impact on implementation efforts when proposing a new classification involving important costs and organization for health care systems.

## Conclusion

KT is guided by models, such as the Knowledge to Action Framework,^4^ that identify key stakeholders, settings, champions, and actions that will influence uptake of research into practice. By collaborating across working groups charged with generating evidence-based recommendations for a new TBI classification system, our K2P group was able to identify targeted groups and behaviors that should be leveraged to facilitate routine use of the proposed CBI-M pillars and retrospective classification systems into practice. The efforts of this working group align with the National Academies of Science, Engineering, and Medicine *Traumatic Brain Injury: A Roadmap for Accelerating Progress*^18^ report that calls for a new classification system, advancement of learning systems for improved TBI care, and for agencies to accelerate collaboration and impact of advancing TBI knowledge and practice. Embedding objective identification of key stakeholders, target settings, and priority actions into these efforts is an essential step for successful practice change, and a critical component in the broader vision to reduce the burden of TBI. This is a first step in identifying implementation priorities, and ultimate success is conditional on an iterative process that assesses opportunities, challenges, stakeholders, and champions throughout the implementation process.

## Transparency, Rigor, and Reproducibility Summary

This survey-based study drew upon established principles of implementation science, specifically Integrated KT and the Knowledge to Action framework. The survey instrument and prioritization process were based upon previously published protocols/instruments and the survey instrument is reproduced in full in the Appendices. The study was not pre-registered. The survey response rate was 59% (40/67). The survey was not designed to be representative as it sought specific content-area knowledge from a defined group of academics across several TBI classification working groups. Strengths and limitations of the methodology are described within the article.

## Appendix

### Appendix A: Copy of Survey

#### Translating TBI classification working group knowledge into practice

Dear TBI Classification Working Group colleagues,

Thanks for taking the time to fill in this short survey which has been developed by the Knowledge to Practice (K2P) Working Group. This Working Group comprises seven researchers who specialise in methods to ensure that health policy and practice is based on best-available health research knowledge so that it can benefit patients.

The survey is designed to enable your Working Group to document ideas about ‘who needs to do what differently’ to implement a new TBI classification framework. Please consider your Working Group’s focus and/or proposed recommendations in completing this short survey. We’ve made it short so that you can set aside a small amount of time at the end of each of your monthly meetings to complete the survey.

Filling the survey out will help us to collate standardized data across all working groups. Ultimately this will be used to build a ‘long list’ of implementation ideas which we can prioritize in advance of the January meeting. We expect this process to be undertaken in November—December. Therefore it will be important for you to complete surveys in your September and October meetings.

We will assign a representative from the Knowledge to Practice Working group to liaise with your working group to address any questions you have about translating your ideas into practice. The representative will also reflect your emerging survey data back to the group so it can be refined and address any questions you have about knowledge translation methods.

**Q1. Please select your Working Group**

Clinical/symptoms (1)

Imaging biomarkers (2)

Blood based biomarkers (3)

Psychosocial and environmental factors (4)

Retrospective classification (5)

**Q2a. Which of the groups listed below are target audiences for your group’s recommendations**—**that is, those who are not familiar with the TBI classification project but will need to change their practices based on the recommendations of your Working Group?** *Click all that apply*

• Allied healthcare workers (1)
• Athletic trainers (2)
• Community healthcare providers (3)
• Community physicians (4)
• Community sport volunteers (5)
• Critical care physicians (6)
• ED physicians (7)
• Funding bodies (8)
• Hospital Administrators (10)
• Industry (e.g. medical technology or device companies) (11)
• Insurers (12)
• Neurologists (13)
• Neuropsychologists (14)
• Neurosurgeons (15)
• Nurses (16)
• Pharmacists (17)
• Policy Makers (18)
• Pre-hospital care practitioners (19)
• Radiologists (20)
• Rehabilitation physicians (21)
• Representative medical professional society (specify) (22)
• Research professional society (specify) (23)
• Researchers (24)
• Respiratory therapists (25)
• Patient/caregivers associations (26)
• Schools (27)
• Social service providers (28)
• Other (please specify) (9) ___________________

*Carry Forward Selected Choices-Entered Text from “Q2a. Which of the groups listed below are target audiences for your group’s recommendations*—*that is, those who are not familiar with the TBI classification project but will need to change their practices based on the recommendations of your Working Group? Click all that apply”*

**Q2b. From your list, please select up to three groups that will be most impacted by your work-that is, those that will have to change their practices the most.** *Select a minimum of one and a maximum of three*

*Carry Forward Selected Choices-Entered Text from “Q2a. Which of the groups listed below are target audiences for your group’s recommendations*—*that is, those who are not familiar with the TBI classification project but will need to change their practices based on the recommendations of your Working Group? Click all that apply”*

**Q2c. From your list, please select up to three groups that will be most resistant to changing their practices.** *Select a minimum of one and a maximum of three*

**Q3a. In what settings will changes need to happen based on the recommendations of your Working Group?** *Click all that apply*

• Community settings, e.g., sporting organizations (3)
• Government research funding organization/department (4)
• Government policymaking organization/department (5)
• Community-based health settings, e.g., physician (1)
• Hospital administration (2)
• Critical care (hospital) (6)
• Emergency Department (hospital) (7)
• Imaging/Radiology (hospital) (8)
• Intensive Care Unit (hospital) (9)
• Laboratory (hospital) (10)
• Neurosurgery (hospital) (11)
• Rehabilitation (hospital) (12)
• Ward (hospital) (13)
• Insurance companies (14)
• Non-government research funding organizations (15)
• Schools (16)
• Scientific publishers/publications (17)
• Universities and colleges (research, teaching, training) (18)
• Technology companies (19)
• Other (please specify) (20) ___________________

*Carry Forward Selected Choices-Entered Text from “Q3a. In what settings will changes need to happen based on the recommendations of your Working Group? Click all that apply”*

**Q3b. From your list, please select up to three settings that will be most impacted by your work-that is, those that will have to change their practices the most.** *Select a minimum of one and a maximum of three*

**Q4. Please describe a specific recommended change to TBI classification practices** based on the discussions and analysis of your Working Group by answering the question:

**“[who?] needs to [do what differently?] in [what setting?]”**

where

**[who]** is the target group that needs to change their behaviour-for example *“athletic trainers”*;

**[do what differently]** is the recommended change based on your Working Group-for example *“conduct a head injury assessment after a head knock”*; and

**[setting]** is where you want the change to happen-for example *“community sport”*

This creates the following knowledge to action statement:

*“****athletic trainers*** *need to* ***conduct a head injury assessment after a head knock*** *in* ***community sport****”*

In the table below, just list the ‘who’, the ‘what they need to do differently’ and the ‘setting’

**Table:**

	Write “who” here (1)	Write “what they need to do differently” here (2)	Write the “setting” here (3)
Recommended Change (1)			

**Q4a. Please describe up to 10 further specific recommended changes to TBI classification practices using the same format:**

**[who]** is the target group that needs to change their behavior-for example *“athletic trainers”*;

**[do what differently]** is the recommended change based on your Working Group-for example *“conduct a head injury assessment after a head knock”*; and

**[setting]** is where you want the change to happen-for example *“community sport”*

**Q5. [OPTIONAL] Which of the groups listed below are potential change champions for your group’s recommendations**—**that is, those who are already familiar with the TBI classification project and could actively promote proposed practice changes?** *Click all that apply.*

• Advocacy organization (specify if able) (1)
• Community organization (specify if able) (2)
• Consumer representative organization (specify if able) (3)
• Community healthcare facility or provider (specify if able) (4)
• Prehospital healthcare facility or provider (specify if able) (5)
• Hospital healthcare facility or provider (specify if able) (6)
• Rehabilitation healthcare facility or provider (specify if able) (7)
• Industry (e.g., medical technology or device company—specify if able) (8)
• Insurance organization (specify if able) (9)
• Media organization (specify if able) (10)
• Peer-reviewed journal publisher (specify if able) (11)
• People with lived experience and their families/caregivers (12)
• Policymaking organization (specify if able) (13)
• Representative medical professional society (specify if able) (14)
• Research professional society (specify if able) (15)
• Other (specify) (16) ________________________

**Q6.** [OPTIONAL] Please use this space to document any questions you would like to ask the Knowledge to Practice Working Group. These can be in relation to this survey and/or more general questions about knowledge translation.

### Appendix B: Guiding Framework for K2P Working Group: Knowledge to Action Framework

**NOTES:** Much of our work centers on the left side of the model-we obtained information from our K2P surveys on settings, stakeholders, champions, and priority behaviors for each set of recommendations. In each working group break out session, note if there are additional facilitators, barriers, adaptations that may inform how to best implement these recommendations. Our initial survey findings were not exhaustive, so getting additional information in real time from stakeholders beyond just the working groups is important*. Below are areas to take notes to add to our findings, and also some discussion questions you can consider posing to the groups as appropriate.*

**NOTES**

Stakeholders:

Affected Settings:

Important Change Champions:

Resources Needed:

Priority Actions/Behaviors:

Additional Observations/Discussion Points:

### Potential Discussion Questions

How might knowledge about these proposed recommendations need to be adapted to increase uptake/use in local settings?

What are some potential barriers to routine implementation of these recommendations in specific practice settings?

Besides the practice settings we identified for this group, are there other settings that should be considered?

What are potential facilitators to implementation of these recommendations?

What are some important resources that would be needed to implement this set of recommendations?

Is there specific technical assistance or guidance that may be needed to increase routine use of these recommendations at practice sites?

Are there additional stakeholder groups that could serve as important stakeholders and/or change champions that we haven’t identified yet for this set of recommendations?

What are some short term, high priority areas that would need to be addressed first to facilitate use of these proposed recommendations? Who would need to be involved in this short term work?

What are some longer term initiatives that should be considered for broader implementation of these recommendations and additional work in this area? Who would need to be involved in this long term work?
